# Novel dietary FemTech based on dietary reference intakes for premenstrual and menstrual disorders: a pilot open-label randomized controlled trial of dietary intervention

**DOI:** 10.1186/s12905-026-04382-6

**Published:** 2026-03-09

**Authors:** Jun Iimura, Naohisa Shobako, Masahiro Yagibashi, Atsushi Nakajima, Shintaro Fujii, Takuo Nakazeko, Yukio Hirano, Futoshi Nakamura, Keiko Honda

**Affiliations:** 1 Future Food Research & Development Division, Nissin Foods Holdings Co., Ltd, Tobukimachi, Hachioji, Japan; 2 TES Holdings Co., Ltd, Higashi Ueno, Tokyo, Japan; 3 Ueno-Asagao Clinic, Higashi Ueno, Tokyo, Japan; 4 Laboratory of Medicine Nutrition, Kagawa Nutrition University, Chiyoda, Sakado, Saitama Japan

**Keywords:** Randomized control trial, Open-label trial, Premenstrual disorder, Optimized Nutri-Dense Meals

## Abstract

**Background:**

We developed novel Optimized Nutri-Dense Meals (Opti meals) for women based on Japanese dietary reference intakes (DRIs). We aimed to determine the effects of Opti meals on premenstrual disorder (PMD).

**Method:**

This pilot study was an open-label randomized controlled trial. One hundred women aged 20–45 years with PMD were enrolled in the study and randomly divided into two groups: habitual diet and intervention groups. In the intervention group, two meals per day for three menstrual cycles were replaced with Opti meals based on the Japanese DRIs. The Menstrual Distress Questionnaire (MDQ) and the Japanese version of the Daily Records of Severity of Problems Short-Form version (J-DRSP) served as the primary outcome measures. Sleep quality and several saliva and serum parameters were set as secondary outcomes.

**Result:**

The intervention group had lower MDQ total scores during the premenstrual (median [Q1–Q3]: 42.0 [23.8–64.0] versus 67.0 [44.0–90.5], *p* < 0.01) and menstrual (34.0 [23.0–53.0] versus 67.0 [35.0–76.5], *p* < 0.01) phases, as well as lower final J-DRSP total scores (17.25 [13.73–19.90] versus 22.0 [16.60–28.50], *p* < 0.01) than the habitual diet group.

Some significant differences were observed in the sleep quality test; however, no significant differences were found in saliva or serum parameters at the end of the trial.

**Conclusion:**

Optimal meals based on the Japanese DRI may improve PMD symptoms.

**Trial registration:**

UMIN000052973, https://center6.umin.ac.jp/cgi-open-bin/ctr_e/ctr_view.cgi?recptno=R000060324 (3/12/2023).

**Supplementary Information:**

The online version contains supplementary material available at 10.1186/s12905-026-04382-6.

## Introduction

Premenstrual disorder (PMD) is a serious issue for women across cultures, and is a comprehensive concept encompassing both physical and mental symptoms [1]. PMD is not an official diagnostic category but a conceptual term commonly used for research context [2]. In clinical practice, diagnosis of premenstrual syndrome (PMS) or premenstrual dysphoric disorder (PMDD) is based on guidelines. PMS is characterized by a constellation of cyclic physical or mood‑related symptoms that emerge in the luteal phase and resolve shortly after the onset of menstruation. These symptoms may include irritability, bloating, mood swings, lethargy, breast tenderness, or anxiety [3]. In contrast, PMDD (Premenstrual Dysphoric Disorder) is a formal psychiatric diagnosis and is included in the Diagnostic and Statistical Manual of Mental Disorders, Fifth Edition [4]. These disorders share the common feature of involving both physical and psychological symptoms, with PMDD characterized in particular by severe emotional symptoms [5].

Both pharmaceutical and non-pharmaceutical interventions have been investigated for managing PMD. Hormonal contraceptives are a pharmacological option; however, long-term use raises concerns regarding adverse effects [6]. Moreover, taking such drugs during Ramadan has been reported to increase the risk of cerebral venous thrombosis [7], and psychological resistance remains a major global challenge [8]. Consequently, non-pharmaceutical strategies have received considerable attention. For instance, the association between PMD and obesity is well established [9, 10]. Previous observational studies have also linked eating habits to PMD [11, 12]. Specifically, a Western diet appears to aggravate PMD [13, 14]. Therefore, maintaining a balanced diet is crucial for managing PMD.

How can a balanced diet be maintained? Supplementation with the lacking nutrients may be an option. For instance, Ahmadi reported that zinc supplementation reduced PMD symptoms [15], and Tartagni reported the same for vitamin D [16]. However, some studies have reported that vitamin D supplementation does not reduce PMD symptoms [17]. Moreover, vitamin B1 supplementation did not show a significant effect in a previous randomized controlled trial (RCT) [18]. As summarized by Carlini, there is insufficient evidence to recommend improvements through interventions with a single nutrient [19]. Furthermore, a meta-analysis combining RCTs on multiple vitamins failed to demonstrate significant efficacy [20].

Therefore, overall dietary interventions and balanced nutrition may represent the next target frontier. Traditional eating patterns, such as the Chinese [21] and Middle Eastern [14] diets, have been cited as beneficial examples; however, they are difficult to replicate because of their unique ingredients [22]. Optimized Nutri-Dense Meals (Opti meals) may address this limitation. This dietary intervention is based on 33 nutrients specified in Japan’s dietary reference intakes (DRIs) rather than specific ingredients [22]. Opti meals encompass a wide variety of dishes, ranging from Western to Chinese menus, and have demonstrated benefits for visceral fat [22], blood pressure [23], blood glucose metabolism [23], and frailty [24]. Here, we modified Opti meals based on the Japanese DRI to meet the nutritional requirements of adult women (Table 1). In this study, we aimed to investigate the effects of Opti meals on PMD.

**Table 1:** Limit of each nutritional component per portion of the tested meal

		/480 kcal	
	Unit	Lower limit	Upper limit
Protein	g	18.9	24.0
Fat	g	10.7	16.0
SFA	g		3.73
n-3PUFA	g	0.64	
n-6 PUFA	g	3.20	
Carbohydrate	g	60.0	78.0
Fiber	g	6.1	
Salt equ.	g /meal		3.0
K	mg	727	
Ca	mg	233	393
Mg	mg	108	
P	mg	291	472
Ir	mg	3.1	7.9
Zn	mg	3.2	6.3
Cu	mg	0.26	1.10
Mn	mg	1.2	1.7
I	μg	38	409
Ser	μg	9	71
Cr	μg	3	79
Mo	μg	9	94
RAE	μg	262	425
VD	μg	2.5	15.7
VE	mg	2.0	133.8
VK	μg	44	
VB1	mg	0.41	
VB2	mg	0.47	
Nia	mg	4.4	12.6*
VB6	mg	0.41	8.66
VB12	μg	0.7	
FA*	μg	116	142
PA	mg	1.75	
Bio	μg	14.5	
VC	mg	29	
Ile	mg	396	
Leu	mg	773	
Lys	mg	594	
Sulphur-containing amino acids	mg	297	
Aromatic amino acids	mg	495	
Thr	mg	297	
Trp	mg	79	
Val	mg	515	
His	mg	198	

SFA: Saturated fatty acid, PUFA: Poly unsaturated fatty acid, Salt equ.: salt equivalent, K: Potassium, Ca: Calcium, Mg: Magnesium, P: Phosphorus, Ir: Iron, Zn: Zinc, Cu: Copper, Mn: Manganese, I: Iodine, Se: Selenium, Cr: Chromium, Mo: Molybdenum, RAE: Retinol active equivalent, VD: Vitamin D, VE: Vitamin E, VK: Vitamin K, VB1: Vitamin B1, VB2: Vitamin B2, Nia: Niacin equivalent, VB6: Vitamin B6, VB12: Vitamin B12, FA: Folic acid, PA: Pantothenic acid, VC: Vitamin C, Ile: Isoleucine, Leu: Leucine, Lys: Lysine, Thr: Threonine, Trp: Tryptophan, Val: Valine, His: Histidine

* Applies only to additives and does not include amounts derived from food ingredients

## Materials and methods

This study was designed and conducted following the Consolidated Standards of Reporting Trials 2025 Guidelines. A complete copy of the checklist is provided in Supplementary Figure S1.

### Study design

**Fig. 1:**
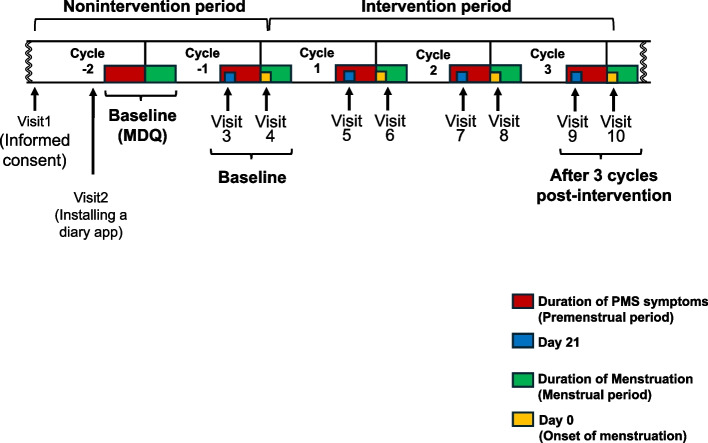
Scheme of the trial. This study was designed based on the participants’ menstrual cycles. The baseline values for the MDQ and other parameters prior to the intervention were based on measurements obtained at the time shown in the figure. The intervention period was set to three menstrual cycles

This open-label, randomized controlled trial was conducted according to the principles of the Declaration of Helsinki. The ethics review board of Ueno Asagao Clinic (approval no. 2023–34) approved this trial in accordance with the ethical guidelines for human research (Ministry of Education, Culture, Sports, Science, and Technology; Ministry of Health, Labor, and Welfare, Japan). This trial was registered with the UMIN Clinical Trials Registry (UMIN000052973). After receiving an explanation of the study, the women who agreed to participate voluntarily signed an informed consent agreement without coercion. The trial scheme is shown in Fig. 1.

### Study population

Participants were recruited through a website from November 14 to December 5, 2023. Those who wanted to participate in the trial were invited to the Ueno Asagao Clinic (Tokyo, Japan), where the study details and potential risks were explained, written informed consent was obtained, and screening was performed.

The inclusion criteria were as follows: (1) healthy women aged 20 to < 45 years; (2) presence of PMD symptoms, defined as unpleasant mental or physical manifestations lasting 3–10 days before menstruation and easing or disappearing at the start of menstruation; (3) menstrual cycles of 25–38 days with fluctuations of ≤ 6 days [25]; (4) regular lifestyles; (5) written informed consent after a full explanation of study purpose and procedures; (6) ability to attend the examination on the specified date; and (7) approval for participation by the principal investigator (medical doctor).

The exclusion criteria were as follows: (1) current disease under pharmacological treatment; (2) regular use of hormones (control pills) or other drugs; (3) history or presence of mental illness, sleep disorders, hypertension, diabetes, dyslipidemia, or serious diseases; (4) history or presence of serious hepatic, renal, cardiac, pulmonary, gastrointestinal, or hematological disorders; (5) habitual medication in the past month for disease treatment (excluding cold or headache remedies); (6) drug or food allergies; (7) difficulty continuing the test food during the study; (8) irregular eating habits (missing breakfast, lunch, or dinner) pronounced food selectivity; (9) daily excessive alcohol consumption; (10) regular intake of foods for specified health uses, foods with functional claims, or health foods (unless suspended during the study); (11) anticipated lifestyle changes during the study (e.g., long-term travel) or potential inability to attend clinic visits because of relocation; (12) non-iPhone users or difficulty wearing the provided Apple Watch throughout the day except when bathing (an iPhone is required to record sympathetic nervous system activity); (13) inability to record basal body temperature and use mobile phone apps daily; (14) inability to store or consume the test food appropriately (e.g., no freezer or microwave oven); (15) pregnancy, breastfeeding, or potential pregnancy during the study; (16) participation in another clinical trial or completion of one < 3 months before enrollment; (17) employment of the participant or a family member at an institution that develops, manufactures, or sells health or functional foods; and (18) any other condition deemed inappropriate by the principal investigator.

### Randomization

Participants were randomly assigned to two groups according to the stratified block randomization method, stratified by age, body mass index, total score on the short version of the Japanese Daily Records of Severity of Problems (J-DRSP), and results of the Menstrual Distress Questionnaire (MDQ). Values at or below the median were coded as 1, and those above the median as 2; thus, an array was generated for each participant. A computer program (Visual Studio 2022, Redmond, WA, USA) randomly produced six group-number patterns (ABAB, AABB, ABBA, BABA, BAAB, and BBAA), and the participants were allocated to the corresponding stratification groups. Following randomization, we confirmed that the groups did not differ significantly in the Oguri-Shirakawa-Azumi (OSA) sleep-questionnaire outcomes. Regarding energy intake, while strict accuracy could not be guaranteed, we examined the possibility of balancing the results of the brief self-administered diet history questionnaire (BDHQ) [26].

### Interventions

The trial was conducted in Tokyo, Japan, between January 11 and May 29, 2024. The test meals were prepared as frozen lunch boxes and delivered to the homes of participants allocated to the test group (Opti group). During the trial, participants replaced their lunch and dinner with the test meal. Participants in the habitual diet group were instructed to continue their habitual diet. Both groups were required to take photos of their meals and upload them to the diary app for 3 days before and after the trial. Additionally, participants were asked to take a picture of any leftover food and post it on the app if they could not finish the test meal.

All test meals contained 33 nutrients within the ranges specified in Table 1. These ranges were established based on the Japanese DRIs with minor modifications. Figure S2 provides an example of a lunch box accompanied by instructions for lunch and dinner, and Table S3 lists the corresponding nutrient content. All 31 menus are summarized in Supplementary Table S1. Meals could be consumed in any sequence. Although snack intake was unrestricted, participants were instructed to avoid overeating. All participants were required to wear an Apple Watch daily throughout the trial. Additionally, they were asked to complete daily app recordings of their basal body temperature; breakfast, snack, and alcohol intake; and exercise duration. Participants in both groups were instructed to maintain their pre-trial habits (including exercise, breakfast, and indulgences), except for the test foods.

### Outcomes

The primary outcomes were the MDQ and J-DRSP scores. Secondary outcomes included OSA-MA, saliva markers (progesterone, testosterone, and 17β-estradiol), serum allopregnanolone, a questionnaire on perceived physical changes (e.g., skin and hair condition), quality of life measured by Apple Watch, body composition, and defecation condition. Moreover, we measured physiological and clinical indicators and administered a questionnaire regarding the test meal. Safety was evaluated based on the number of adverse events, interviews with the principal investigator, and participant diaries.

### Procedures

PMS severity was assessed using the MDQ, as described previously [27, 28]. The questionnaire comprises 47 items covering eight PMS subscales. Participants rated the intensity of 48 symptoms on a 4-point scale, and the total score was used as the final score; however, item 44 (“Changed in food preference”) was excluded from the total because our intervention was dietary intervention. The participants completed the printed version of the MDQ at home and submitted it during their hospital visits. The MDQ was completed 14 days after menstruation onset. Using recall, the participants reported their conditions before, during, and after the menstrual cycle. The premenstrual period was defined as 21 ± 3 days after the previous menstruation, menstruation as the day (± 3 days) bleeding began, and the post-menstrual period as 14 days after the onset. Measurement timing followed that of a previous study [29].

PMDD severity was evaluated using the short-form version of the J-DRSP, previously reported by Ikeda et al.; the validity and reliability of this short and Japanese-translated version have been demonstrated [30]. Participants answered on a 6-point scale regarding the intensity of the eight questions, looking back on the day; the responses were given daily during the trial. We calculated and evaluated total psychological and physical scores [30], as well as the average number of days prior to menstruation onset.

The quality of sleep was evaluated using OSA sleep inventory, Middle Aged and Aged (MA) version (OSA-MA) [31]. Participants completed the survey immediately upon awakening at each clinical visit, during both the premenstrual and menstrual periods.

Saliva was collected on the morning of the clinical visit days from Visit 3 to Visit 10. Upon awakening, participants were required to gargle and collect approximately 1.5 mL of saliva after at least 10 min. The saliva samples were frozen until biomarker measurements were performed. 17-β-estradiol, progesterone, and testosterone were measured using enzyme-linked immunosorbent assay kits (Salimetrics LLC, CA, USA).

During the trial period, the participants were required to wear the provided Apple Watch. The Apple Watch recorded the sympathetic nervous system activity during the trial.

Allopregnanolone was quantified in purified serum collected during visits 3, 5, 7, and 9. After the addition of the internal standard (AP-d5), tert-butyl methyl ether (MTBE) was introduced, and the mixture was separated into aqueous and organic phases. The organic phase was dried in a centrifugal evaporator, and the residue was redissolved in methanol, diluted with purified water, and purified using an OASIS MAX cartridge (Waters, MA, USA). The eluate was removed by centrifugal evaporation and derivatized by adding 50 μL of a mixture containing 80 mg 2-methyl-6-nitrobenzoic anhydride, 20 mg 4-dimethylaminopyridine, 40 mg picric acid, and 10 μL triethylamine in 1 mL acetonitrile, followed by reaction at room temperature for 30 min. The reaction mixture was purified using an InertSep SI cartridge, and allopregnanolone was determined by LC–MS as previously reported [32].

The following additional parameters were measured: white blood cells, red blood cells, hemoglobin, hematocrit value, mean corpuscular volume, mean corpuscular hemoglobin, mean corpuscular hemoglobin concentration, platelet count, total protein, creatinine, uric acid, alanine aminotransferase, aspartate aminotransferase, γ-glutamyl transpeptidase, alkaline phosphatase, lactate dehydrogenase, total cholesterol, low density lipoprotein cholesterol, high density lipoprotein cholesterol, total bilirubin, blood glucose level, hemoglobin a1c, triglycerides, sodium, potassium, chloride, calcium, magnesium, ferrum, insulin, glycoalbumin, c reactive protein, ferritin, total iron binding capacity, and unsaturated iron binding capacity. Approximately 13 mL of blood was collected from each participant during each hospital visit.

Urinary urobilinogen, hidden blood, bilirubin, ketone body qualitative test, glucose qualitative test, protein, pH, and specific gravity were also measured. Participants provided approximately 10 mL of urine in the morning of their visit.

Blood pressure was measured using a TM-2580 instrument (A&D Company, Tokyo, Japan). After resting, the cuff was wrapped around the upper right arm, and measurements were taken twice while maintaining the cuff at the heart level. The second result was adopted as the final blood pressure reading.

Basal body temperature was measured using a basal thermometer (C531; Terumo Corporation, Tokyo, Japan). The participants were instructed to remain still upon awakening, and their basal temperatures were measured while they were still in bed. The temperature was measured by holding the thermometer against the tongue and keeping the mouth closed. During temperature measurement, breathing through the mouth was prohibited, and participants were asked to record their temperature in their diaries.

Participants’ dietary records were evaluated using two approaches. First, the photo-usage method was employed to estimate nutrient intake for the 3 days preceding the intervention and the 3 days preceding its end. Participants photographed every meal (breakfast, lunch, dinner, snacks, and alcoholic beverages) for 3 consecutive days with their mobile phones, and the nutrient content was calculated using Healthy Maker Pro (Mushroom Soft Co., Ltd., Okayama, Japan). Second, the BDHQ was used to estimate nutrient intake during the month before screening.

### Statistical analysis

The parameters analyzed in this study are presented as mean±standard deviation (SD) or median with interquartile range (Q1–Q3). Although this was the first trial of Opti meals to determine PMD, we referred to previous research evaluating the effects of other foods on PMD [33]. Based on a previously reported effect size (d = 0.7290), a power of 90%, and an alpha of 5%, we calculated that 41 participants were required. Therefore, we enrolled 50 participants per group, allowing for potential dropouts. Efficacy analyses were conducted on the per-protocol set, whereas safety analyses were conducted using a modified intention-to-treat approach (full analysis set).

Primary results were analyzed nonparametrically. The Mann–Whitney U test was used to compare these values. For all two-sided tests, the significance level was set at 5% (*p* < 0.05). OSA was analyzed similarly. Other continuous parameters, including serum and saliva levels, were examined using two-tailed Student’s t-tests, adopting a significance threshold of 5% (*p* < 0.05). All analyses were performed using IBM SPSS Statistics for Windows, version 26.0 (IBM Corp., NY, US). Efficacy was assessed solely by comparing the final measurement data at the end of the intervention period.

In addition to this pre-specified primary analysis, a Generalized Linear Mixed Model (GLMM) was conducted as a post hoc exploratory analysis, with the aim of examining the robustness of the findings while accounting for repeated measurements and individual-level variability.

In the GLMM, participants were treated as random effects, whereas group (Opti vs. habitual diet), time (menstrual cycles), and their interaction (group × time) were included as fixed effects. The model was fitted using the GLIMMIX procedure in SAS 9.4 (SAS Institute Inc., Cary, NC, USA), assuming a normal distribution. Within-subject residual covariance was specified as variance components (VC). This model allowed us to estimate the longitudinal intervention effect while appropriately considering within-subject correlations.

## Results

### Participants

**Fig. 2:**
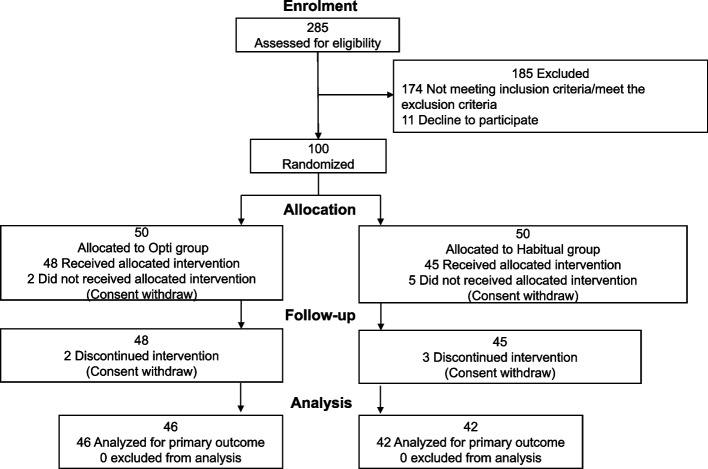
CONSORT flow diagram of the study participants. This flowchart indicates the selection scheme used. In total, 100 participants were selected from 285 entries and divided into the Opti and habitual diet groups. Each group comprised 50 participants. Before the intervention began, two participants in the Opti group and five in the habitual diet group did not receive the allocated intervention (withdrew before initiation). During the intervention period, two participants from the Opti group and three from the habitual diet group withdrew their consent. All 88 participants who completed the trial (46 in the Opti group and 42 in the habitual diet group) were included in the primary outcome analyses

Overall, 285 participants were included in this study. One hundred participants were enrolled and randomly assigned to the Opti or Habitual diet groups (Fig. 2). The demographic characteristics representing the baseline values (measured timing is shown in Fig. 1) of each group are presented in Table 2. The overall dropout rate was 12% (12/100), and the reason for dropping out was unrelated to the intervention or trials. Table 3 presents the average nutrient intake over the 3 days preceding the intervention and the 3 days before its termination. No significant difference was observed in energy intake between the two periods (before and after). However, significant differences were observed in multiple nutrients, including macro- and micronutrients,, such as fiber, calcium, copper, and vitamin D content. During the test period, the participants’ sympathetic nervous system activity was recorded using an Apple Watch, except during bathing, and no significant intergroup differences (data not shown).

**Table 2:** Participant characteristics

Item		Unit	Habitual			Opti		
	Age	years	34.7	±	7.5	34.2	±	6.8
	Body weight	kg	53.5	±	7.4	54.2	±	7.9
	BMI	kg/m^2^	21.6	±	2.9	21.3	±	2.8
DRSP								
	Physical score		12.2		(9.8–17.3)	12.0		(9.3–15.6)
	Psychological score		11.5		(8.6–13.6)	9.5		(6.9–12.2)
	Total score		23.5		(19.3–30.0)	21.3		(16.0–27.5)
MDQ								
	Pain		11		(7.0–14.0)	11		(7.0–15.0)
	Concentration		14		(6.0–18.0)	11		(5.0–15.3)
	Behavioral changes		11		(9.0–12.3)	10		(6.0–12.3)
	Autonomic reactions		2		(0.8–6.0)	2		(0.0–4.0)
	Water retention		9		(6.0–10.0)	8		(6.0–10.0)
	Negative affect		15		(11.8–20.0)	13		(7.8–18.0)
	Arousal		3		(2.0–4.3)	3		(0.0–5.0)
	Control		3		(0.8–7.0)	1		(0.0–4.3)
	Total Score		73		(49.8–85.3)	55		(38.3–81.5)
OSA-MA								
	Sleepiness on rising		42.7		(38.4–50.0)	45.7		(36.4–52.1)
	Initiation and maintenance of sleep		44.4		(35.5–53.2)	43.7		(35.8–49.5)
	Frequent dreaming		49.6		(35.9–58.4)	58.4		(40.8–58.4)
	Refreshing in rising		43.3		(42.4–51.6)	47.2		(42.4–51.2)
	Sleep length		50.1		(42.5–53.1)	50.1		(34.9–51.5)
Biomarkers								
	17-β-estradiol	pg/mL	1.6	±	0.8	1.3	±	0.5
	Progesterone	pg/mL	394.7	±	250.1	379.3	±	340.2
	Testosterone	pg/mL	126.0	±	52.8	124.1	±	52.2
	Allopregnanolone	pg/mL	452.1	±	400.7	550.7	±	532.8
Other parameters								
	Triglyceride	mg/dL	73	±	41.1	73	±	29.0
	LDL cholesterol	mg/dL	111	±	33.4	110	±	26.2
	HDL cholesterol	mg/dL	72	±	14.6	75	±	15.2
	Fasting blood glucose	mg/dL	87	±	9.1	85	±	8.1
	HbA1c	%	5.1	±	0.24	5.1	±	0.26
	SBP	mmHg	107	±	11.8	105	±	12.9
	DBP	mmHg	68	±	9.2	65	±	8.8
	Iron	μg/dL	94	±	36.2	87	±	37.6
	Ferritin quantification	ng/mL	28.1	±	24.17	27.6	±	29.41

Each value is expressed as the mean ± SD or median (Q1-Q3)

*Habitual* Habitual diet group, *Opti* Opti meal group, *DRSP* Daily Record of Severity of Problems, *MDQ* Menstrual Distress Questionnaire, *OSA-MA* OSA sleep inventory MA version, *BMI* Body mass index, *SBP* Systolic blood pressure, *DBP* Diastolic blood pressure

**Table 3:** Average nutrient intake during the test duration

Item	Unit	Group	Before			p	After			p
Energy	kcal/day	Habitual	1493	±	369.6	0.2969	1451	±	322.4	0.8533
		Opti	1413	±	350.8		1440	±	265.9	
Protein	g/day	Habitual	55.8	±	14.2	0.9535	57.3	±	14.9	0.5074
		Opti	56.0	±	16.8		59.1	±	10.3	
Fats	g/day	Habitual	57.2	±	16.8	0.1222	57.8	±	18.4	0.0001
		Opti	51.2	±	19.0		43.6	±	13.6	
SFA	g/day	Habitual	17.9	±	6.1	0.0696	18.3	±	7.2	< 0.0001
		Opti	15.5	±	6.3		11.9	±	5.4	
MUSFA	g/day	Habitual	21.4	±	6.3	0.0950	21.3	±	7.2	< 0.0001
		Opti	18.9	±	7.5		13.6	±	5.2	
PUSFA	g/day	Habitual	10.8	±	3.5	0.6776	11.3	±	3.9	0.0262
		Opti	10.4	±	4.3		12.8	±	2.5	
Carbohydrate	g/day	Habitual	198.1	±	50.7	0.293	185.7	±	40.0	0.0419
		Opti	187.9	±	40.2		202.3	±	35.7	
Soluble dietary fiber	g/day	Habitual	5.9	±	1.9	0.3619	5.4	±	1.6	< 0.0001
		Opti	5.6	±	1.9		8.3	±	1.1	
Insoluble dietary fiber	g/day	Habitual	8.1	±	2.3	0.2626	7.3	±	2.2	0.4014
		Opti	7.5	±	2.8		7.6	±	1.9	
Sodium	mg/day	Habitual	2946	±	783.6	0.7902	2950	±	718.6	0.0001
		Opti	2901	±	797.0		2412	±	494.6	
Potassium	mg/day	Habitual	1841	±	501.5	0.6813	1782	±	511.8	< 0.0001
		Opti	1790	±	650.2		2382	±	414.6	
Calcium	mg/day	Habitual	340	±	136.4	0.7202	380	±	131.4	< 0.0001
		Opti	330	±	134.7		638	±	113.1	
Magnesium	mg/day	Habitual	182	±	46.4	0.4617	193	±	55.4	< 0.0001
		Opti	191	±	64.7		284	±	36.2	
Phosphorus	mg/day	Habitual	785	±	203.4	0.8734	828	±	210.7	0.0001
		Opti	777	±	234.4		988	±	164.2	
Iron	mg/day	Habitual	5.5	±	1.3	0.2884	5.8	±	1.9	< 0.0001
		Opti	5.9	±	2.2		9.4	±	1.2	
Zinc	mg/day	Habitual	7.1	±	2.6	0.7031	6.5	±	1.9	< 0.0001
		Opti	6.9	±	2.2		10.3	±	1.4	
Copper	mg/day	Habitual	1	±	0.3	0.4015	1	±	0.3	0.0028
		Opti	1	±	0.3		1	±	0.2	
Manganese	mg/day	Habitual	2	±	0.7	0.9202	2	±	0.6	< 0.0001
		Opti	2	±	0.8		4	±	0.6	
VA	RAE	Habitual	411	±	333.9	0.2773	455	±	493.4	< 0.0001
		Opti	351	±	164.6		921	±	127.7	
VD	μg/day	Habitual	3.8	±	3.6	0.0804	4.6	±	2.8	< 0.0001
		Opti	5.2	±	4.0		11.5	±	3.1	
VE	mg/day	Habitual	6.0	±	1.9	0.7556	6.0	±	2.0	< 0.0001
		Opti	5.9	±	2.5		9.0	±	1.6	
VK	μg/day	Habitual	157	±	98.6	0.3920	148	±	83.5	< 0.0001
		Opti	181	±	157.9		226	±	65.4	
VB1	mg/day	Habitual	0.80	±	0.3	0.9987	0.80	±	0.2	< 0.0001
		Opti	0.80	±	0.3		1.39	±	0.2	
VB2	mg/day	Habitual	0.9	±	0.3	0.9326	0.9	±	0.3	< 0.0001
		Opti	0.9	±	0.4		2.0	±	0.3	
VB6	mg/day	Habitual	1.00	±	0.3	0.8533	1.00	±	0.4	< 0.0001
		Opti	0.98	±	0.4		1.39	±	0.2	
VB12	μg/day	Habitual	4.2	±	3.8	0.1822	5.6	±	4.1	0.8420
		Opti	5.2	±	3.8		5.4	±	2.6	
FA	μg/day	Habitual	219	±	72.0	0.9300	211	±	78.3	< 0.0001
		Opti	221	±	103.7		438	±	47.1	
PA	mg/day	Habitual	4.66	±	1.3	0.4716	4.44	±	1.3	< 0.0001
		Opti	4.43	±	1.6		6.52	±	1.1	
VC	mg/day	Habitual	72	±	32.1	0.0885	57	±	26.0	< 0.0001
		Opti	59	±	36.8		135	±	24.5	

The detailed explanation about the duration was cited in the results section

*Habitual* Habitual diet group, *Opti* Opti meal group, *SFA* Saturated fatty acids, *MUSFA* Monounsaturated fatty acids, *PUSFA* Poly unsaturated fatty acids, *VA* Vitamin A, *RAE* Retinol active equivalent, *VD* Vitamin D, *VE* Vitamin E, *VK* Vitamin K, *VB1* Vitamin B1, *VB2* Vitamin B2, *FA* Folic acid, *PA* Pantothenic acid, *VC* Vitamin C

#### Effectiveness

**Fig. 3:**
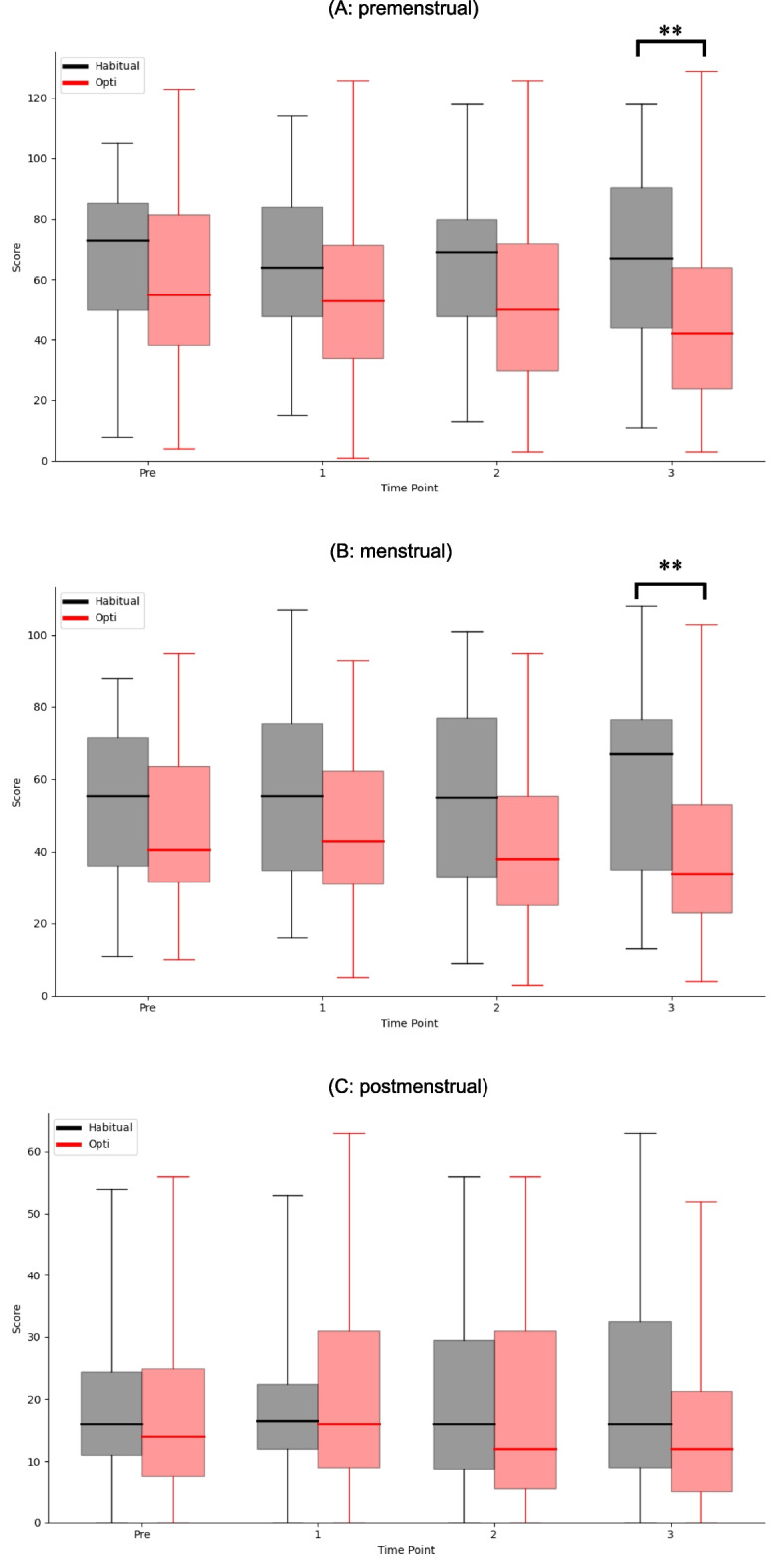
Result of MDQ. (**A** measured in the premenstrual era, **B** measured in the menstrual era, **C** measured in the post-menstrual era). The horizontal axis indicates the menstrual cycle. The vertical axis represents the scores. Higher scores indicate greater pain. For each boxplot, the central horizontal line represents the median. The upper and lower edges of the box correspond to the first (Q1) and third (Q3) quartiles, respectively, indicating the interquartile range (IQR). Whiskers extend to the minimum and maximum observed values. * *p* < 0.05, **p < 0.01. The MDQ score at premenstrual timing was significantly lower in the Opti group than in the habitual diet group during the second and third cycles. At menstruation, the same trend was observed from the first to the third cycle. No significant differences were observed between the groups after menstruation

Figure 3 depicts the total MDQ score for the primary outcomes, whereas Table S4 lists the MDQ subscores. Higher scores indicate greater pain. The MDQ was evaluated three times for each menstrual cycle: pre-menstruation, menstruation, and post-menstruation. In the third cycle, which was the last measurement after the intervention, the total score of the Opti group was significantly lower than that of the habitual diet group.

The median [Q1–Q3] values in the Opti group versus habitual diet group were 42.0 [23.8–64.0] versus 67.0 [44.0–90.5] at the premenstrual stage, 34.0 [23.0–53.0] versus 67.0 [35.0–76.5] at the menstrual stage, and 12.0 [5.0–21.3] versus 16.0 [9.0–32.5] at the postmenstrual stage (*p* = 0.002, *p* = 0.004, and *p* = 0.098, respectively).

Significant differences were also shown in the “VI negative affect” and “VIII control” subscale at menstrual stage. The score was 5.0 [2.0–10.0] vs13.0 [4.5–16.0] (*p* = 0.0016) and 0.0 [0.0–3.0] vs 2.0 [0.0–5.5] (*p* = 0.0364), respectively.

**Fig. 4:**
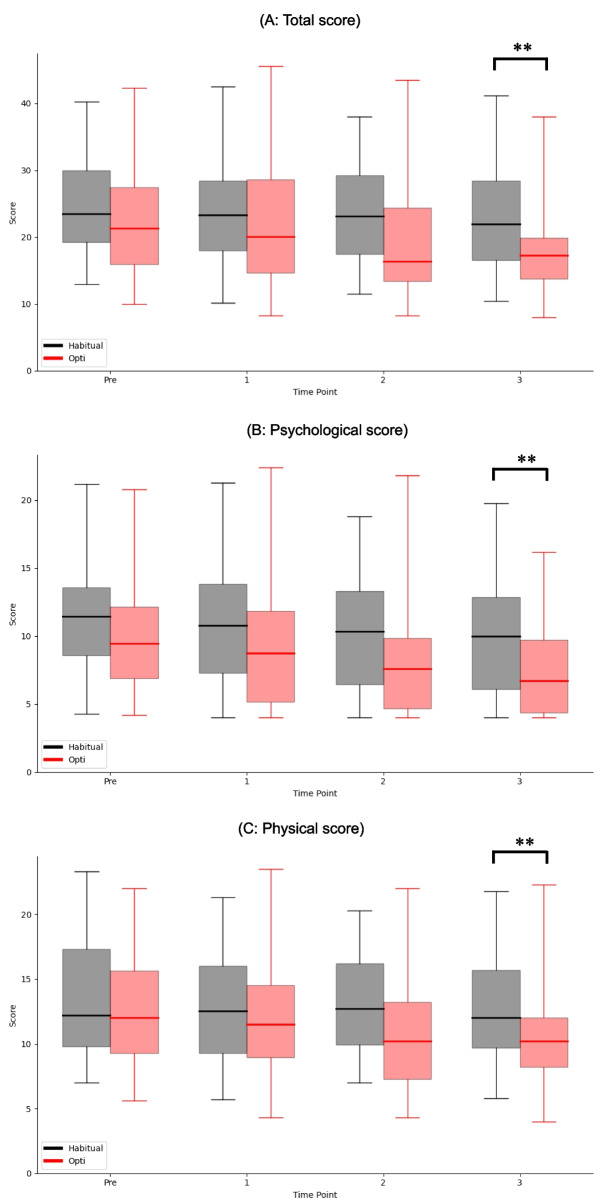
Result of J-DRSP. (**A** total score, **B** psychological score subscale, **C** physical score subscale). The horizontal axis indicates the menstrual cycles. The vertical axis represents the scores. A higher scores indicates greater pain. For each boxplot, the central horizontal line represents the median. The upper and lower edges of the box correspond to the first (Q1) and third (Q3) quartiles, indicating the interquartile range (IQR). Whiskers extend to the minimum and maximum observed values.* *p* < 0.05, **p < 0.01. The total score was significantly lower in the Opti group than in the habitual diet group during the second and third menstrual cycles. Psychological and physical scores were significantly lower in the Opti group than in the habitual diet group at the third cycle

Figure 4 presents the J-DRSP results for each measurement point (A, total score; B, psychological score; C, physical score). Higher scores indicate greater pain. The figure shows that the total and sub-scores at the final measurement were significantly lower in the Opti group than in the habitual diet group. The total scores at final measurement were 17.25 [13.73–19.90] and 22.00 [16.60–28.50] in Opti and habitual diet groups, respectively (*p* = 0.004). Psychological score was 6.70 [4.38–9.73] and 10.00 [6.10–12.85] in Opti and habitual diet groups, respectively (*p* = 0.006). Physical score was 10.20 [8.23–12.00] and 12.00 [9.70–15.70] in Opti and habitual diet groups, respectively (*p* = 0.007).

**Fig. 5:**
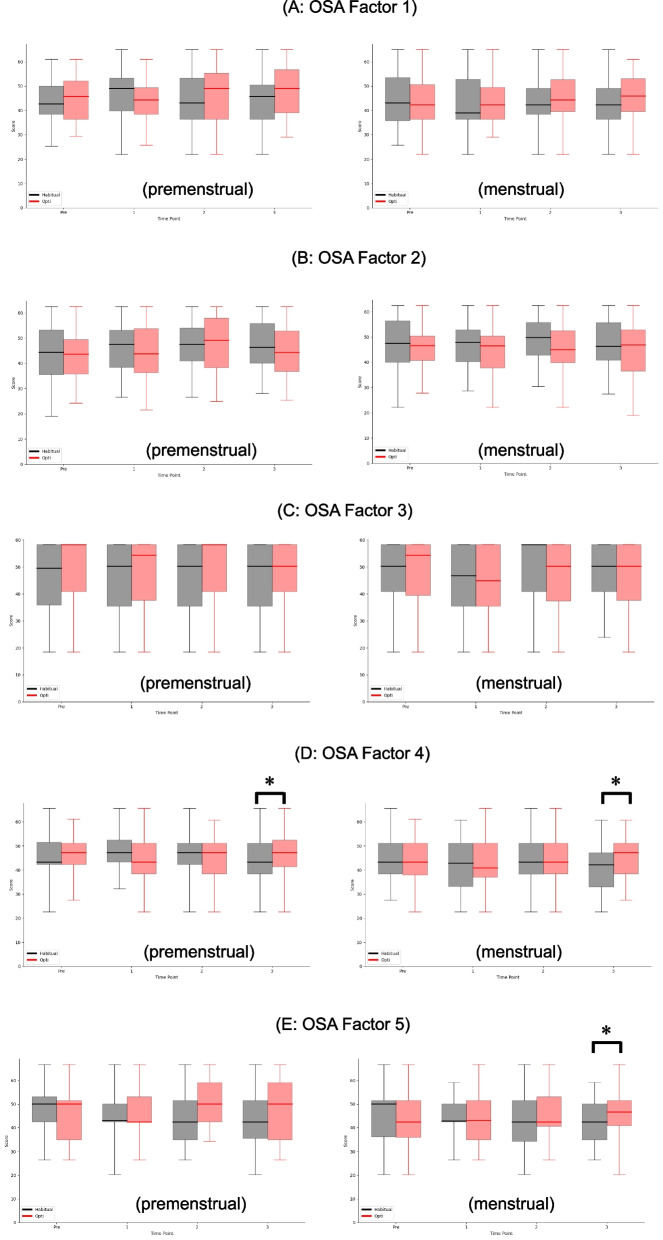
Results of OSA. Higher scores on this questionnaire indicate better sleep quality for the corresponding items. For each boxplot, the central horizontal line represents the median value. The upper and lower edges of the box correspond to the first quartile (Q1) and third quartile (Q3), indicating the interquartile range (IQR). Whiskers extend to the minimum and maximum observed values. * p<0.05, **p<0.01. Panels A–E correspond to the five factors of the OSA-MA: (A) factor1; sleepiness on rising, (B) factor2; initiation and maintenance of sleep, (C) factor3; frequent dreaming, (D) factor4; refreshing on rising, and (E) factor5; sleep length. In the third menstrual cycle, the Opti group scored higher than the habitual diet group for factors 4 and 5

Figure 5 shows the OSA results (A–E), a secondary outcome, for which higher scores denote better sleep quality. At 21 days (final measurement), the Factor 4 score was significantly higher in the Opti group than in the habitual diet group (47.24 [41.38–52.50] versus 43.29 [38.42–51.18], Fig. 5D). At the onset of menstruation, the Opti group displayed significantly higher scores than the habitual diet group for Factors 4 (47.24 [38.42–51.18] versus 42.17 [33.06–47.24], Fig. 5D) and 5 (46.67 [40.94–51.53] versus 42.50 [34.86–50.14], Fig. 5E). Figure S3 summarizes the saliva and serum parameters; no significant differences between the intervention and habitual diet groups were observed at the final measurement.

We also conducted a post-hoc analysis to confirm the robustness of intervention effect even if accounting for individual variability by GLMM. As shown in Table 4, significant time × group interactions (*p* < 0.05) were observed for the MDQ in the premenstrual and menstrual phases, and for the J-DRSP total and physical scores.

**Table 4:** Result of GLMM

(A: MDQ)					
Menstrual cycle phase	Intergroup differences (*p*-Value)	Time (*p*-Value)	Time × Group (*p*-Value)	Estimated difference	Confidence interval for the difference
Premenstrual	0.0459	0.0021	0.0006	−10.8927	−21.5822~−0.2032
Menstrual	0.0131	0.2738	0.0106	−11.8857	−21.2122 ~ −2.5593
Postmenstrual	0.5800	0.6115	0.2695	−1.5581	−7.1307 ~ 4.0145

(B: J-DRSP)					
Item	Intergroup differences (*p*-Value)	Time (*p*-Value)	Time × Group (*p*-Value)	Estimated difference	Confidence interval for the difference
Total score	0.0519	< 0.0001	0.0437	−3.0907	−6.2076 ~ 0.02617
Psychological score	0.0428	< 0.0001	0.1646	−1.7421	−3.4266~−0.05765
Physical score	0.1026	0.0007	0.0199	−1.3517	−2.9806~ 0.2773

#### Safety

The principal investigator determined that the test meal had no adverse effects. Overall, 75 and 73 adverse events were reported in the habitual and Opti groups, respectively. A list of all reported adverse events is provided in Supplementary Table S2.

## Discussion

This study demonstrated that menstruation-related symptoms can be mitigated through dietary interventions designed according to the Japanese DRI. Although previous trials have examined supplementation with individual or combined vitamins and minerals for PMD, the evidence remains inconclusive [19]. Therefore, we addressed this issue by intervening in the entire diet, balancing both micro- and macronutrients. The following discussion focuses on the two principal changes observed after the intervention.

First, we discuss changes in the primary outcomes, which are directly linked to menstruation-related symptoms. PMS encompasses both physical and mental symptoms, the latter formally termed PMDD [34], and is sometimes identified as a continuum representing a more severe form of PMD [34]. PMS symptoms were assessed using the MDQ [35]. PMS symptoms were assessed by the MDQ [36, 37], whereas the DRSP was originally developed for PMDD [37, 38]. Therefore, we evaluated the effect of Opti meals on PMD using both the MDQ and J-DRSP. As illustrated in Fig. 3, Opti meals appeared to lower MDQ scores both before and during menstruation (Fig. 3 A, B). The MDQ comprises 47 items allocated to eight categories. PMS severity was judged based on the eight subscale scores and the total score. Although yoga has been shown to improve total MDQ scores [38], dietary interventions have rarely been reported. A few functional foods, including lactoferrin [39], adlay [40], and probiotics [41], improved individual MDQ subscales but failed to affect the total scores. Conversely, a negative correlation between saturated fatty acid (SFA) or carbohydrate intake and the MDQ score has been reported in retrospective studies [42]. As shown in Table 1, Opti meals fulfilled various nutrient requirements and limited SFAs intake based on DRI recommendations. Unlike past “single material” interventions, Opti meals improved MDQ in multiple aspects.

Our J-DRSP findings for PMDD paralleled those obtained with the MDQ. In a previous RCT, Al Kiyumi reported that a “healthy diet” failed to ameliorate PMDD [43]. In that trial, food-based guidelines were applied, and the participants’ ingredient intake was tightly controlled. As described previously [22], there are two types of guidelines: food-based and DRIs. Food-based guidelines are advantageous because they are easy to reproduce, being free from nutritional calculations. Nonetheless, the correct management of multiple micronutrients, such as vitamins and minerals, is difficult. DRI-based Opti meals overcome this limitation, potentially explaining the observed improvement in J-DRSP.

Only a limited number of RCTs on dietary interventions have demonstrated improvements in PMD and PMDD. As described above, no dietary intervention has been reported to improve the total MDQ score. *Dioscorea esculenta*, a type of yam, was reported to improve DRSP but not MDQ simultaneously [44]. Thus, to the best of our knowledge, this is the first report of simultaneous enhancement of MDQ and DRSP. Moreover, our findings suggest the importance of continuing a DRI-based diet rather than opting for dietary supplements. Although no significant difference was observed, a certain baseline difference in the MDQ is noteworthy for interpreting the results.

Second, we discuss the secondary outcomes, which include sleep quality and various blood parameters associated with PMD. Cross-sectional studies have documented a significant association between sleep quality and PMD [45, 46]. Among several reported methods for measuring sleep quality, we selected the OSA-MA [47], which evaluates five dimensions and has been employed in RCTs of dietary interventions. Some interventions, such as specific teas [48, 49], produced no changes in any dimension, whereas others modified one or two factors [50–52]. As depicted in Fig. 4A-E, we detected significant improvements in Factors 4 and 5, representing post-sleep refreshment and perceived sleep duration, respectively. Nonetheless, the causal relationship between improved sleep and improved PMD remains unclear. However, the causal relationship between improved sleep and improved PMD and sleep quality [53]. Future research should clarify the causal pathways and identify the functional nutrients in Opti meals. The Mediterranean diet is commonly proposed as a healthy eating pattern. Nevertheless, its effects on the OSA sleep questionnaire remain unclear. Similar considerations can be applied to the Nordic and DASH diets. Therefore, our study appears to be the first to report the effects of dietary interventions on sleep quality.

In contrast, Opti meals did not affect saliva or serum hormone levels (Figure S3). Notably, these hormones are reported to be key factors in PMD progression [19], and supplementation with some of them has been attempted to treat PMD [54, 55]. Most crucially, fluctuations in these parameters have been proposed as the underlying cause of PMD [56]. Although the present study focused only on quantity, we plan to examine fluctuations in these parameters in future research. Furthermore, we plan to assess biomarkers at post-intervention time points in subsequent studies.

Previously, we obtained observational data, which showed that providing Opti meals in employee cafeterias improves not only employees' visceral fat area and blood pressure levels but also their working productivity [57]. Based on these results, in future studies, we plan to evaluate the effect of Opti meals on the work productivity of women experiencing PMD. Moreover, the impact of Opti meals on public health can also be evaluated by providing these meals through public meal services.

This study had a few limitations. First, the primary outcome selected as a proxy for PMD relied on participants’ subjective evaluations. The inherent nature of this disorder makes it difficult to establish objective criteria for PMD, especially PMDD [58]. In our trial, Opti meals improved both the MDQ and J-DRSP, which may confer greater robustness than studies reporting improvement in only one measure. However, this trial did not include participants diagnosed with PMD in medical institutions. In the future, we would like to discuss the effectiveness of our intervention through RCTs specified for such patients. Second, the trial was open-label; because the participants knew their allocation, caution is warranted when interpreting the results. In the future, we plan to use control diets and conduct double-blind RCTs. Third, the mechanisms underlying the effects of Opti meals were not investigated. We plan to examine which nutrients defined in the DRIs are key factors, along with the pathways involved. Since we couldn’t find the changes in the amount of hormones such as progesterone (Fig. S3), underlying mechanism might be determined for the further studies. Furthermore, we would like to measure biomarkers at the post-intervention time point for further study. Notably, neurotransmitters, such as serotonin, have also been reported as key factors in PMD development [59]. Thus, we plan to investigate the association between Opti meals and neurotransmitter fluctuations in the future. Fourth, this trial was designed as a pilot study with several primary outcomes. Future research may require conducting RCTs with a single primary outcome to rigorously evaluate the effectiveness of this novel intervention.

## Conclusions

Our findings demonstrate that Opti meals, comprising 33 nutrients defined by the Japanese DRIs and substituting two daily meals, ameliorated PMD symptoms. In the third menstrual cycle, MDQ scores (median [Q1-Q3]) during both the premenstrual and menstrual phases were significantly lower in the Opti group than in the habitual diet group (premenstrual phase: 42.0 [23.8–64.0] versus 67.0 [44.0–90.5]) and menstrual phase: 34.0 [23.0–53.0] versus 67.0 [35.0–76.5]; *p* < 0.01 and *p* < 0.01, respectively). The total J-DRSP score was also reduced in the Opti group than in habitual diet group (17.25 [13.73–19.90] versus 22.00 [16.60–28.50]; *p* < 0.01), with significant improvements in the psychological (6.70 [4.38–9.73] versus 10.00 [6.10–12.85]; *p* < 0.01) and physical (10.20 [8.23–12.00] versus 12.00 [9.70–15.70]; *p* < 0.01) subscales. Among the secondary outcomes, Opti meals enhanced sleep quality, as measured by the OSA-MA: Factor 4 (post-sleep refreshment) and Factor 5 (perceived sleep length) also improved (*p* < 0.05). In contrast, PMD-related biomarkers, including progesterone, testosterone, 17β-estradiol, and allopregnanolone, remained Collectively, these data underscore the value of maintaining a nutritionally balanced diet in accordance with the DRIs.

## Supplementary Information

Supplementary Material 1. Figure S1: CONSORT2025 checklist.

Supplementary Material 2. Figure S2: Example test meal. This image shows a hamburg steak lunch box provided to the Opti group for consumption during lunch or dinner. The nutritional content of the samples is summarized in Table S3. Figure S3: Results of saliva and serum parameters. * *p*<0.05, **p<0.01. Although some items showed significant differences, no results indicated changes attributable to the intervention.

Supplementary Material 3. Table S1: All menu items of the tested meals and delivery schedules. Table S2: All reported adverse events. S3: Nutritional content of the test food (Hamburg steak lunch box). S4: Results of the MDQ subscale. S5: DRSP results for each item; * *p*<0.05, **p<0.01.***p<0.001.

Supplementary Material 4. S6: Results of additional analysis: the Hodges-Lehmann estimate and its confidence interval for the between-group comparison primary outcomes.

## Data Availability

The researchers presented plans with appropriate methodologies and obtained consent from all co-authors.

## Abbreviations

αTOC: α-Tocopherol
BDHQ: Brief self-administered diet history questionnaire
BMI: Body mass index
BP: Blood pressure
Ca: Calcium
COMB meal: Completely balanced meal
Cu: Copper
Cr: Chromium
DASH: Dietary Approaches to Stop Hypertension
DBP: Diastolic blood pressure
DHA: Docosahexaenoic acid
EPA: Eicosatetraenoic acid
FA: Folic acid
FBG: Fasting blood glucose
Ir: Iron
HbA1c: Hemoglobin A1c
I: Iodine
K: Potassium
LDL-C: Low-density lipoprotein cholesterol
MD: Mediterranean diet
Mg: Magnesium
Mn: Manganese
Mo: Molybdenum
n6FA: N6 fatty acid
n3FA: N3 fatty acid
Nia: Niacin
P: Phosphorus
PA: Pantothenic acid
RAE: Retinol active equivalent
PMD: Premenstrual Disorder
PMDD: Premenstrual Dysphoric Disorder
PMS: Premenstrual Syndrome
SBP: Systolic blood pressure
Se: Selenium
TG: Triglyceride
VB1: Vitamin B1
VB2: Vitamin B2
VB6: Vitamin B6
VB12: Vitamin B12
VC: Vitamin C
VD: Vitamin D
VK: Vitamin K
Zn: Zinc
