# Supplementary material for: Novel dietary FemTech based on dietary reference intakes for premenstrual and menstrual disorders: a pilot open-label randomized controlled trial of dietary intervention
Source: BMC Womens Health. 2026 Mar 9;26:178. doi: 10.1186/s12905-026-04382-6 (PMC13019880; doi:10.1186/s12905-026-04382-6)
Supplement: Supplementary file 2 — Supplementary Material 2. Figure S2: Example test meal. This image shows a hamburg steak lunch box provided to the Opti group for consumption during lunch or dinner. The nutritional content of the samples is summarized in Table S3. Figure S3: Results of saliva and serum parameters. * p<0.05, **p<0.01. Although some items showed significant differences, no results indicated changes attributable to the intervention. [file 12905_2026_4382_MOESM2_ESM.pptx]

## Slide 1
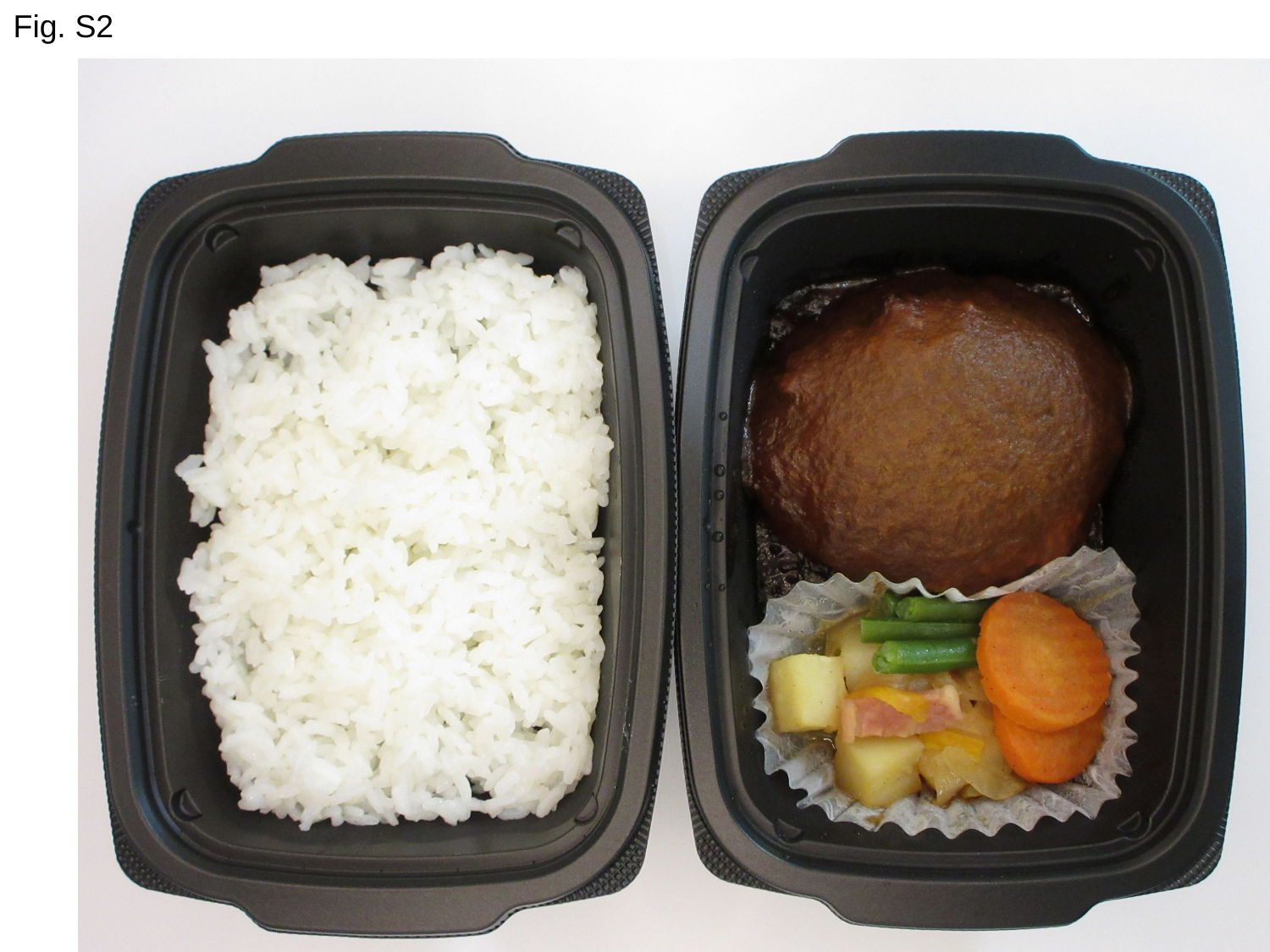

Fig. S2

## Slide 2
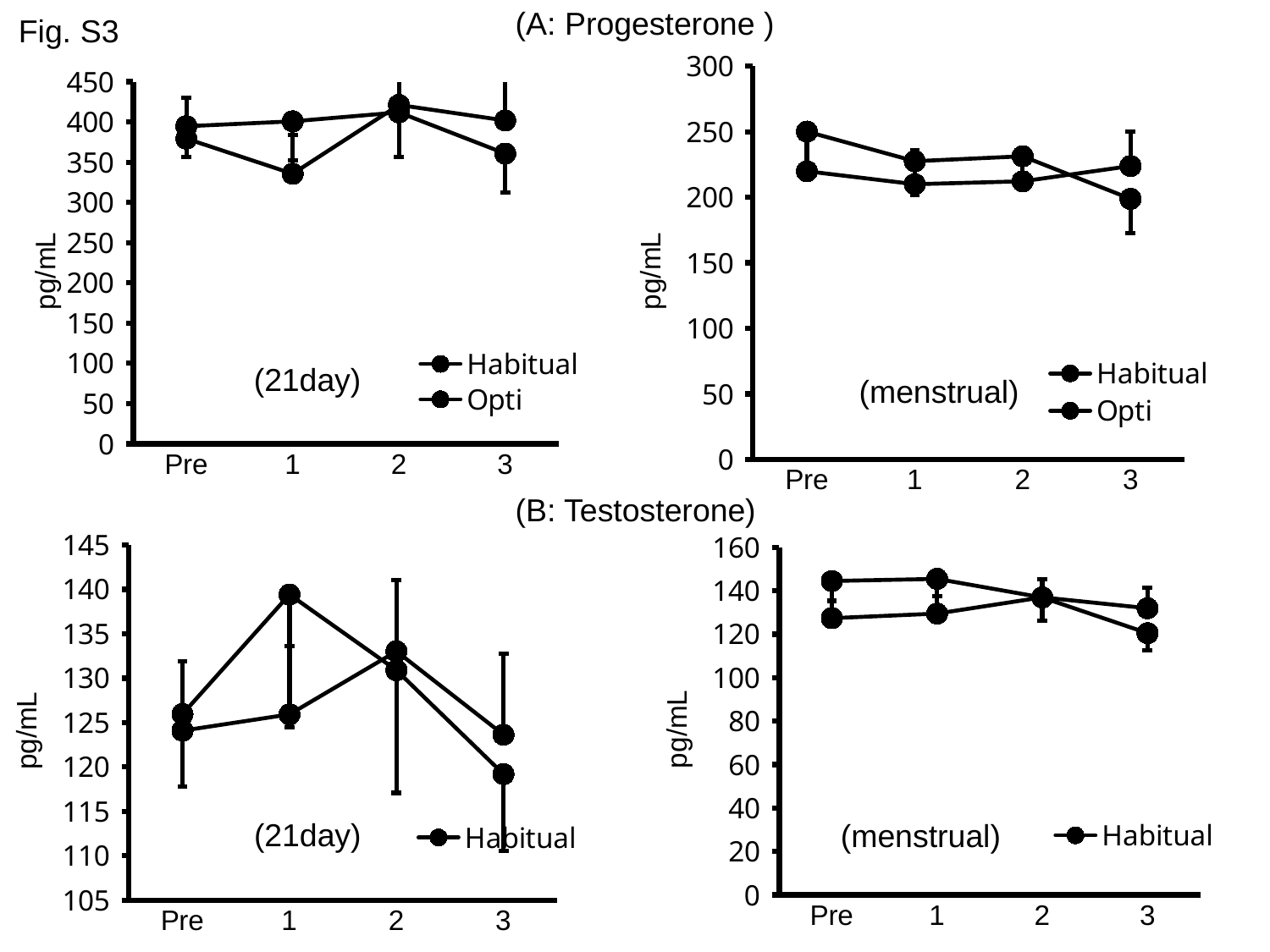

(A: Progesterone )
Fig. S3
### Chart
| Category | Habitual | Opti |
|---|---|---|
| Pre | 250.1558 | 219.8978 |
| 1 | 227.5407 | 210.0103 |
| 2 | 231.3161 | 212.2119 |
| 3 | 198.8592 | 223.8845 |
### Chart
| Category | Habitual | Opti |
|---|---|---|
| Pre | 394.6708 | 379.2714 |
| 1 | 400.7421 | 335.5837 |
| 2 | 411.8556 | 421.0899 |
| 3 | 360.6473 | 401.8035 |(21day)
(menstrual)
(B: Testosterone)
### Chart
| Category | Habitual | Opti |
|---|---|---|
| Pre | 125.9509 | 124.087 |
| 1 | 139.4191 | 125.9202 |
| 2 | 130.8872 | 133.0193 |
| 3 | 119.1897 | 123.632 |
### Chart
| Category | Habitual | Opti |
|---|---|---|
| Pre | 144.4468 | 127.3487 |
| 1 | 145.4223 | 129.4531 |
| 2 | 136.8601 | 136.9308 |
| 3 | 120.445 | 131.9184 |(21day)
(menstrual)

## Slide 3
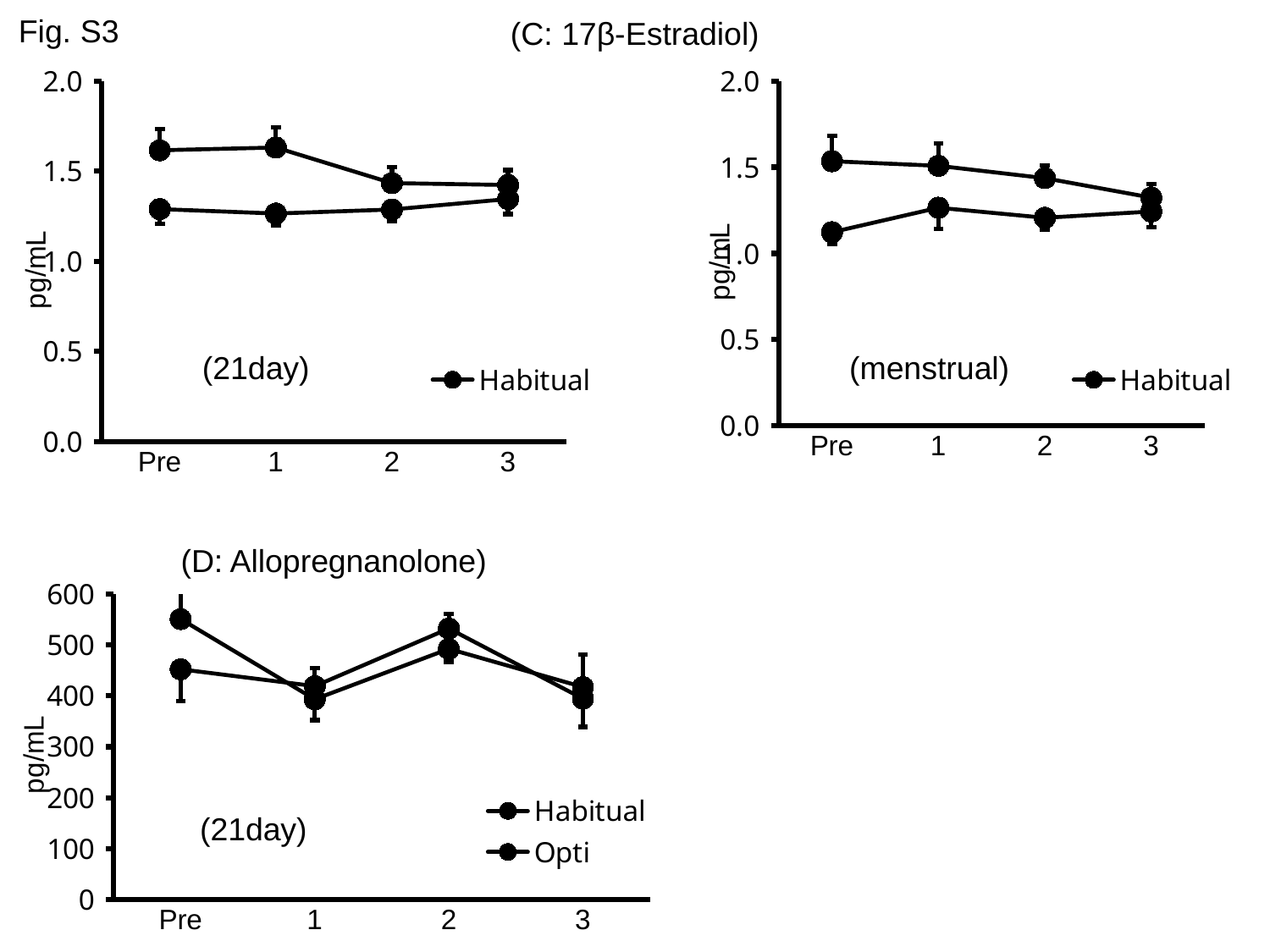

Fig. S3
(C: 17β-Estradiol)
### Chart
| Category | Habitual | Opti |
|---|---|---|
| Pre | 1.6157 | 1.2895 |
| 1 | 1.631 | 1.2648 |
| 2 | 1.4335 | 1.2874 |
| 3 | 1.4232 | 1.3453 |
### Chart
| Category | Habitual | Opti |
|---|---|---|
| Pre | 1.5345 | 1.1227 |
| 1 | 1.5079 | 1.2649 |
| 2 | 1.4365 | 1.2065 |
| 3 | 1.3237 | 1.243 |(21day)
(menstrual)
(D: Allopregnanolone)
### Chart
| Category | Habitual | Opti |
|---|---|---|
| Pre | 452.11 | 550.66 |
| 1 | 419.07 | 393.23 |
| 2 | 532.16 | 492.2 |
| 3 | 394.39 | 417.32 |(21day)
